# Patient and health provider costs of integrated HIV, diabetes and hypertension ambulatory health services in low-income settings — an empirical socio-economic cohort study in Tanzania and Uganda

**DOI:** 10.1186/s12916-021-02094-2

**Published:** 2021-09-10

**Authors:** Tinevimbo Shiri, Josephine Birungi, Anupam V. Garrib, Sokoine L. Kivuyo, Ivan Namakoola, Janneth Mghamba, Joshua Musinguzi, Godfather Kimaro, Gerald Mutungi, Moffat J. Nyirenda, Joseph Okebe, Kaushik Ramaiya, M. Bachmann, Nelson K. Sewankambo, Sayoki Mfinanga, Shabbar Jaffar, Louis W. Niessen

**Affiliations:** 1 Liverpool School of Tropical Medicine, Pembroke Place, Liverpool, L3 5QA UK; 2 The AIDS Support Organisation, Mulago Hospital Complex, Kampala, Uganda; 3 Medical Research Council/Uganda Virus Research Institute & London School of Hygiene and Tropical Medicine Uganda Research Unit, Entebbe, Uganda; 4 National Institutes for Medical Research, Dar es Salaam, Tanzania; 5 Directors Office, Ministry of Health, Community Development, Gender, Elderly and Children, Kampala, Uganda; 6 School of Public Health, Muhimbili University of Health and Allied Sciences, Dar es Salaam, Tanzania; 7 Non-Communicable Diseases Control Programme, Ministry of Health, Kampala, Uganda; 8 London School of Hygiene & Tropical Medicine, Keppel Street, London, WC1E 7HT UK; 9 Hindu Mandal Hospital, Dar es Salaam, Tanzania; 10 Norwich Medical School, University of East Anglia, Norwich, UK; 11 Makerere University College of Health Sciences, Kampala, Uganda; 12 Department of International Health, Johns Hopkins Bloomberg School of Public Health, Baltimore, MD USA

**Keywords:** HIV control, Diabetes, Hypertension, Integrated care, Primary level, Economics, Efficiency, Vulnerable populations, Multi-morbidity

## Abstract

**Background:**

Integration of health services might be an efficient strategy for managing multiple chronic conditions in sub-Saharan Africa, considering the scope of treatments and synergies in service delivery. Proven to promote compliance, integration may lead to increased economies-of-scale. However, evidence on the socio-economic consequences of integration for providers and patients is lacking.

We assessed the clinical resource use, staff time, relative service efficiency and overall societal costs associated with integrating HIV, diabetes and hypertension services in single one-stop clinics where persons with one or more of these conditions were managed.

**Methods:**

2273 participants living with HIV infection, diabetes, or hypertension or combinations of these conditions were enrolled in 10 primary health facilities in Tanzania and Uganda and followed-up for up to 12 months. We collected data on resources used from all participants and on out-of-pocket costs in a sub-sample of 1531 participants, while a facility-level costing study was conducted at each facility. Health worker time per participant was assessed in a time-motion morbidity-stratified study among 228 participants. The mean health service cost per month and out-of-pocket costs per participant visit were calculated in 2020 US$ prices. Nested bootstrapping from these samples accounted for uncertainties. A data envelopment approach was used to benchmark the efficiency of the integrated services. Last, we estimated the budgetary consequences of integration, based on prevalence-based projections until 2025, for both country populations.

**Results:**

Their average retention after 1 year service follow-up was 1911/2273 (84.1%). Five hundred and eighty-two of 2273 (25.6%) participants had two or all three chronic conditions and 1691/2273 (74.4%) had a single condition. During the study, 84/2239 (3.8%) participants acquired a second or third condition. The mean service costs per month of managing two conditions in a single participant were $39.11 (95% CI 33.99, 44.33), $32.18 (95% CI 30.35, 34.07) and $22.65 (95% CI 21.86, 23.43) for the combinations of HIV and diabetes and of HIV and hypertension, diabetes and hypertension, respectively. These costs were 34.4% (95% CI 17.9%, 41.9%) lower as compared to managing any two conditions separately in two different participants. The cost of managing an individual with all three conditions was 48.8% (95% CI 42.1%, 55.3%) lower as compared to managing these conditions separately.

Out-of-pocket healthcare expenditure per participant per visit was $7.33 (95% CI 3.70, 15.86). This constituted 23.4% (95% CI 9.9, 54.3) of the total monthly service expenditure per patient and 11.7% (95% CI 7.3, 22.1) of their individual total household income. The integrated clinics’ mean efficiency benchmark score was 0.86 (range 0.30–1.00) suggesting undercapacity that could serve more participants without compromising quality of care. The estimated budgetary consequences of managing multi-morbidity in these types of integrated clinics is likely to increase by 21.5% (range 19.2–23.4%) in the next 5 years, including substantial savings of 21.6% on the provision of integrated care for vulnerable patients with multi-morbidities.

**Conclusion:**

Integration of HIV services with diabetes and hypertension control reduces both health service and household costs, substantially. It is likely an efficient and equitable way to address the increasing burden of financially vulnerable households among Africa’s ageing populations.

Additional economic evidence is needed from longer-term larger-scale implementation studies to compare extended integrated care packages directly simultaneously with evidence on sustained clinical outcomes.

**Supplementary Information:**

The online version contains supplementary material available at 10.1186/s12916-021-02094-2.

## Introduction

The burden of non-communicable diseases (NCDs) has risen rapidly in Africa, and these diseases typically affect younger working populations than seen in developed countries [1–5]. Diabetes and hypertension alone are probably responsible for more than 2 million deaths a year on the continent [6]. Africa has a continuing high burden of HIV infection. Up until now, health services for HIV infection are organised in stand-alone clinics, separate from the rest of the health system, with separate channels for drug and diagnostic procurement and separate funding. The achievements of HIV programmes are impressive, with over 60% of people living with HIV infection in East and Southern Africa in regular care [7, 8]. In contrast, health care services for diabetes and hypertension, which are also often organised separately, are patchy, with only about 10–20% of people with these conditions estimated to be in care [9–11].

We evaluated the provision of an integrated health care service, via a ‘one-stop’ clinic, for a cohort of people living with HIV infection, diabetes, hypertension, or any combination of these conditions in Tanzania and Uganda at primary and secondary level to assess the acceptability of integrated care if compliance in HIV control was not affected negatively as was feared by country stake holders and health workers [12–15]. However, chronic conditions may lead households into catastrophic expenditure and deeply into poverty [16]. There are few data on the overall economic impact relating to provision of diabetes and hypertension services in Africa and to our knowledge none in relation to one-stop integrated care that specifically includes patients with multiple conditions [17–20].

We aimed to (1) measure the health service attendance and resource use, total time spend per patient and associated treatment costs in our cohort, as well as the additional out-of-pocket (OOP) costs per participant; (2) assess the relative efficiency of integrating services for HIV infection, diabetes and hypertension treatment; and (3) assess the imminent budgetary consequences of managing multi-morbidity in one-stop integrated clinics.

## Methods

### Study design and participants

The cohort study of integration of chronic care services, known as the Management of Chronic Conditions in Africa (MOCCA) study, has been described elsewhere [12]. In brief, 2416 individuals with either HIV, diabetes, hypertension, or combinations of these conditions were invited to join an integrated care clinic and 2273 were enrolled from 5th August 2018 and 21st May 2019, followed-up until the 30th of January 2020 [12–14]. The clinic delivered a single model of care, to patients regardless of condition. All patients were seen by the same clinical staff and shared a single reception, waiting areas and pharmacy, and systems for tracking and follow-up of patients, recording of clinical notes, counselling, appointments, patient cards and registrations. Where not available for patients with diabetes and hypertension, these were set up from new and aligned with those available for people living with HIV. At the onset, missing equipment and materials were supplemented. A refresher training for 1 day was carried out, also at study onset. Next, once-a-month continuous medication sessions were held on chronic conditions with health facility staff as part of their regular schedule. These aimed to ensure standardised clinical management in all three conditions. A small buffer supply of drugs was available to facilities in the event of there being interruptions to drug supplies.

We compared the total costs per patient with multi-morbidities to the sum of treating the same conditions separately.

### Patient selection

People were asked to become a participant in the cohort study on voluntary basis, as we assessed if integrated care were to be of ‘no harm’. The number of patients with diabetes or with multiple conditions was limited; hence, all persons presenting with diabetes or with two or three of target conditions were invited consecutively to join. The numbers of people in care with HIV infection or with hypertension was large and so we sampled systematically, usually every 20th patient. In two small clinics, we enrolled patients known to study staff and invited them to the study. We excluded patients that were severely ill and needed referral to secondary care levels and patients that would not continue to live in the area.

### Socio-economic study

**Fig. 1:**
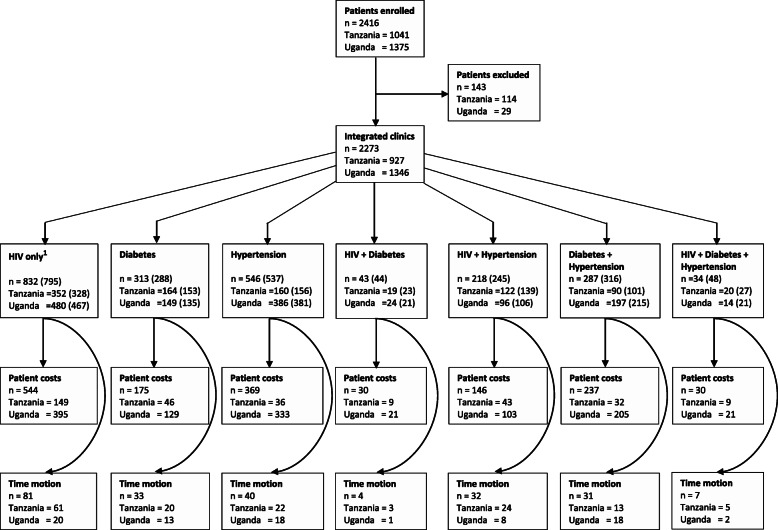
Number of patients enrolled in each sub-study by diagnosis status

The embedded economic sub-study adopted both health service and patient perspectives, that is, a broad societal perspective. A costing study of delivering services for HIV, diabetes and hypertension was conducted in each participating facility. Individual resource use was recorded in all participants in the overall clinical questionnaire. We collected detailed patient cost information on the volume of resources in 1531 patients and their households, shown in Fig. 1, on the second row. This figure shows that in Tanzania 1041 patients were included and in Uganda 1375. The lowest number of participants (below 50) was in the group of participants with both HIV and diabetes and the group with the three target conditions. All other groups had more than 200 participants with the HIV as the largest with 832 participants.

We recorded the diagnostic tests performed and the drugs prescribed, individually. Unit costs for both individual drugs and diagnostic tests were available at the facility level, based on suppliers’ invoices, and where they were not available, local market prices were adopted. Unit costs and specific diagnostic tests are shown in Tables A1 and A2, respectively, in the Additional file 1. The direct and indirect costs were prospectively measured in the sub-sample of 1531 participants, recruited consecutively. Also, a sub-sample of 228 participants was enrolled randomly in a comorbidity-stratified time-motion study to assess health worker time spent per participant per visit (Fig. 1).

### Collection of data on integrated programme overheads

A standardised costing tool [21] was administered to estimate facility level costs in each of the 10 facilities, both in Uganda and Tanzania. It collected information on numbers and level of facility health staff to estimate salary and related costs of all staff involved in integrated service delivery. This was supplemented with information on numbers of other staff involved in caring for the participant, office supplies, travel expenses, utility bills, medication, medical consumables and supplies, number of laboratory testing, medical equipment, furniture, vehicles and rental space used for client care. Rental costs were based on the total area of the space utilised and the average cost per square metre in each setting. Routine information on total patient numbers at each facility every month was collected to facilitate the attribution of the integrated care service. The supply of drugs and diagnostic tests was variable. Some of the facilities had insufficient drug supply, inadequate functional laboratory equipment to conduct tests and some lacked the equipment and or reagents to conduct some laboratory tests (Table A3 in the Additional file 1). We therefore calculated the health service cost per patient at each facility based on the observed use of resources and assumed a full month’s supply of drugs and diagnostics per each supply round.

### Collection of data on participant-related out-of-pocket costs

We administered a patient cost questionnaire to a sub-sample of participants, sequentially (Fig. 1). Information collected included participant income, total household income, demographic characteristics, medical and nonmedical costs prior to attending to the health care facility and on the day of attendance, loss of earnings due to participants attending a health care facility and details on health care financing such as if the patients had to borrow money or sell valuables to be able to attend the healthcare facility. The participant’s reported monthly income was used to calculate the value of hours lost. In addition, we collected information on the participants’ travel time to the health facility, transport costs, costs of meals and other out-of-pocket payments such as payments for health services and medications.

### Time-motion study involving health workers and participants

In our time-motion study, we recorded the time each type of health facility staff spent providing care and the time that participants spent waiting for each component of health care. The total time spent at the facility by participants was added to their travelling time to estimate productivity losses related to their visit (see annex for further details).

### Statistical analysis

In an ingredient approach to collect resource use data [22], following consolidated health economic evaluation reporting standards (CHEERS) guidelines [23], we calculated total health service cost and out-of-pocket cost per participant by comorbidity category. We assigned costs related to drugs, diagnostics, programme costs (such as staff salaries, administration, furniture and equipment), transport and other out-of-pocket expenses, including total loss of productivity due to time spent visiting the facility. We used non-parametric bootstrapping [24] to assess the uncertainties which were reported as 95% confidence intervals (95% CI). All costs were calculated in 2020 US$ prices. The estimated participant and programme overhead costs for facility-level management of diabetes and hypertension treatment were suboptimal due to shortages of medications and staff. We calculated the actual real-life cost and re-assigned the overheads and personnel costs calculated for the people living with HIV infection to those living with diabetes and hypertension. The number of drug and diagnostic tests was based on the actual reported resources used by the participants.

We present here aggregate and country-based uncertainty distributions of clinical resource use within each subgroup group and across groups. These are determined by the health guidelines used in the training and reinforced by repeat and continuous education sessions. These estimates document the complete range of services cost per individual and allows for a comparison of distributions between the various types of patients, in the diverse settings.

To assess and to benchmark the efficiency of the integrated clinics, we applied an input-output data envelopment analysis (DEA) [25–27] to measure the relative efficiency of HIV clinics, NCD clinics and outpatient departments that manage HIV, diabetes and hypertension in Uganda and Tanzania.

The efficiency of a facility is defined as the ratio of the weighted sum of outputs (total virtual output) to the weighted sum of inputs (total virtual input), with the weights being obtained in favour of each evaluated facility to measure the optimisation of activities processes. Each facility is using multiple inputs to produce multiple outputs. We denote a vector of inputs for so-called decision-making unit (DMUs) as well as a vector of outputs is outputs. The model [27] is formulated and solved for each hospital to obtain its efficiency score, once relative weights associated with the inputs and the outputs are identified and valued, respectively. These weights are calculated in a manner that they provide the highest possible efficiency score for each facility under evaluation [27].

We standardised key outputs given key resource inputs of the facilities. In the computation, we re-scaled the included output metrics between 0 and 1. A score of 1 equals the maximum outputs score observed among the individual clinics, given the input levels. We included the number of participants attended and diagnostic tests, and medicines provided were used as the output benchmarks. The number of key clinical staff was used as input variables, categorised into three groups: clinicians, nurses and other health workers which included laboratory technicians and pharmacists. We excluded the use of health services or patient costs data, as these differ between the two counties. The outputs were specified as (1) the staff workload defined as the number of full-time staff divided by the number of daily participant visits, (2) laboratory tests per participant defined as the number of daily laboratory tests divided by the number of daily participant visits and (3) drug availability defined by the proportion of patients who receive drugs at no costs from the facilities before stock-outs occur.

We used the empirical integration cost estimates in a future forecasting for the two individual country settings, based on DEA results and prevalence-based modelling accounting for shorter survival and including the observed uncertainty ranges of the total cost per patient profile. We conservatively assumed that prevalence of these — multiple — conditions will remain constant and independent over time (see Additional file 1 for per country details). The source of the country population projections is United Nations Population Council [13]. All analyses were conducted using SAS version 9.4 (SAS Institute, Inc., NC). The necessary assumption here is that the estimates from the study need to be representative for similar situations, elsewhere in the two countries. Presently, this cannot be verified, and the estimates need to be viewed at as ‘what-if’ scenarios. The uncertainties in the presented changes are more driven by the uncertainties in the demographic-epidemiological data and included the narrow uncertainty ranges of the collected cost per patient categories. Conservatively, we present the relative changes in the health service cost projections.

## Results

### Study participation

Their retention in care after 1 year follow-up was 1911/2273 (84.1%). At enrolment, 582/2273 (25.6%) participants had 2 or all 3 chronic conditions of study (referred to hereon as multi-morbidity) while the remainder 1691/2273 (74.4%) had a single condition. Over the course of the study, an additional 84/2239 (3.8%) participants acquired a second or third condition. Figure 1 shows the flow diagram of participants in the economic study.

Baseline characteristics for individuals sampled to provide participant out-of-pocket expenses are shown in Table 1. Two-thirds or more were women in each disease category. Overall, half (529/1050) were self-employed and 19% (284/1504) reported no income. Most participants (78%; 1159/1481) used public transport to access health facilities. Strikingly, over a third of the participants (563/1492) left a young child below the age of 6 years at home and 10% (149/1510) asked a family member or friend to accompany them to the health care facility.

**Table 1:** The characteristics of individuals who were enrolled for patient-related costs by disease condition. Values are presented as % (n), with the denominator given by the total count with a response for that characteristic, except for average group age.

Characteristics	Category	Overall	HIV	Diabetes	Hypertension	HIV + diabetes	HIV + hypertension	Diabetes + hypertension	HIV + diabetes + hypertension
Age	Average (range)		**38 (18–78)**	**49 (19–77)**	**56 (27–86)**	**55 (21–85)**			
Gender	Total	**1531**	**544**	**175**	**369**	**30**	**146**	**237**	**30**
	Female	71.5 (1094)	68.0 (370)	67.4 (118)	75.3 (278)	66.7 (20)	70.5 (103)	76.4 (181)	80.0 (24)
	Male	28.5 (437)	32.0 (174)	32.6 (57)	24.7 (91)	33.3 (10)	29.5 (43)	23.6 (56)	20.0 (6)
Highest education level	Total	**1051**	**407**	**138**	**235**	**18**	**90**	**142**	**21**
	No formal education	9.4 (99)	5.9 (24)	12.3 (17)	9.8 (23)	11.1 (2)	7.8 (7)	15.5 (22)	19.0 (4)
	Primary education	61.7 (648)	62.4 (254)	61.6 (85)	62.1 (146)	50.0 (9)	62.2 (56)	61.3 (87)	52.4 (11)
	Secondary education	22.4 (235)	24.6 (100)	18.1 (25)	21.7 (51)	27.8 (5)	26.7 (24)	16.9 (24)	28.6 (6)
	Higher education	6.6 (69)	7.1 (29)	8.0 (11)	6.4 (15)	11.1 (2)	3.3 (3)	6.3 (9)	0.0 (0)
Marital status	Total	**1053**	**407**	**138**	**236**	**18**	**90**	**143**	**21**
	Currently married	51.0 (537)	39.8 (162)	68.8 (95)	57.2 (135)	61.1 (11)	47.8 (43)	58.0 (83)	38.1 (8)
	Separated	15.6 (164)	17.9 (73)	13.0 (18)	17.4 (41)	5.6 (1)	11.1 (10)	14.0 (20)	4.8 (1)
	Widowed	14.9 (157)	9.8 (40)	8.0 (11)	18.6 (44)	11.1 (2)	26.7 (24)	17.5 (25)	52.4 (11)
	Never married	13.6 (143)	25.1 (102)	9.4 (13)	4.7 (11)	16.7 (3)	4.4 (4)	7.0 (10)	0.0 (0)
	Divorced	4.9 (52)	7.4 (30)	0.7 (1)	2.1 (5)	5.6 (1)	10.0 (9)	3.5 (5)	4.8 (1)
Participant’s main occupation	Total	**1050**	**405**	**138**	**236**	**18**	**90**	**142**	**21**
	Self-employed	50.4 (529)	51.1 (207)	51.4 (71)	48.3 (114)	50.0 (9)	51.1 (46)	50.0 (71)	52.4 (11)
	Casual labourer	11.7 (123)	17.3 (70)	5.8 (8)	8.9 (21)	16.7 (3)	6.7 (6)	10.6 (15)	0.0 (0)
	Non-governmental organisation employee	10.2 (107)	11.1 (45)	10.1 (14)	12.7 (30)	16.7 (3)	8.9 (8)	4.9 (7)	0.0 (0)
	Unemployed (able to work)	9.5 (100)	8.6 (35)	10.9 (15)	7.2 (17)	5.6 (1)	7.8 (7)	16.2 (23)	9.5 (2)
	Housewife	7.5 (79)	5.9 (24)	10.9 (15)	6.4 (15)	5.6 (1)	12.2 (11)	7.0 (10)	14.3 (3)
	Unemployed (unable to work)	5.0 (52)	2.2 (9)	2.2 (3)	9.3 (22)	0.0 (0)	7.8 (7)	5.6 (8)	14.3 (3)
	Government employee	2.9 (30)	1.2 (5)	4.3 (6)	4.7 (11)	5.6 (1)	3.3 (3)	2.8 (4)	0.0 (0)
	Retired	2.3 (24)	1.5 (6)	3.6 (5)	2.5 (6)	0.0 (0)	2.2 (2)	2.1 (3)	9.5 (2)
	Student	0.6 (6)	1.0 (4)	0.7 (1)	0.0 (0)	0.0 (0)	0.0 (0)	0.7 (1)	0.0 (0)
Individual who contributes most income in the household	Total	**1525**	**544**	**175**	**363**	**30**	**146**	**237**	**30**
	Self	57.8 (881)	62.5 (340)	52.0 (91)	54 (196)	66.7 (20)	61.0 (89)	53.6 (127)	60.0 (18)
	Spouse	19.4 (296)	19.3 (105)	26.9 (47)	18.5 (67)	20.0 (6)	13.0 (19)	20.7 (49)	10.0 (3)
	Children	13.9 (212)	3.5 (19)	10.9 (19)	22.0 (80)	3.3 (1)	19.9 (29)	23.2 (55)	30.0 (9)
	Brother/sister	4.1 (63)	6.8 (37)	4.6 (8)	2.8 (10)	6.7 (2)	2.1 (3)	1.3 (3)	0.0 (0)
	Other	3.0 (46)	4.4 (24)	2.9 (5)	2.8 (10)	0.0 (0)	2.7 (4)	1.3 (3)	0.0 (0)
	Parent	1.8 (27)	3.5 (19)	2.9 (5)	0.0 (0)	3.3 (1)	1.4 (2)	0.0 (0)	0.0 (0)
Main occupation of the person who contributes most in the household	Total	**770**	**265**	**100**	**177**	**16**	**74**	**123**	**15**
	Self-employed	55.7 (429)	52.5 (139)	56.0 (56)	59.3 (105)	50.0 (8)	51.4 (38)	60.2 (74)	60.0 (9)
	Professional	24.2 (186)	23.4 (62)	26.0 (26)	25.4 (45)	31.3 (5)	23.0 (17)	22.0 (27)	26.7 (4)
	Other	10.0 (77)	13.2 (35)	7.0 (7)	7.9 (14)	0.0 (0)	14.9 (11)	6.5 (8)	13.3 (2)
	Farmer	6.1 (47)	6.4 (17)	7.0 (7)	5.1 (9)	18.8 (3)	2.7 (2)	7.3 (9)	0.0 (0)
	Unemployed	3.0 (23)	4.2 (11)	1.0 (1)	1.1 (2)	0.0 (0)	5.4 (4)	4.1 (5)	0.0 (0)
	Housewife	1.0 (8)	0.4 (1)	3.0 (3)	1.1 (2)	0.0 (0)	2.7 (2)	0.0 (0)	0.0 (0)
Participant’s main income is based on	Total	**1504**	**538**	**171**	**361**	**30**	**140**	**235**	**29**
	Business earnings	23.7 (356)	22.1 (119)	26.9 (46)	22.7 (82)	33.3 (10)	22.9 (32)	24.3 (57)	34.5 (10)
	Daily wage	27.4 (412)	32.7 (176)	26.3 (45)	19.9 (72)	16.7 (5)	25.7 (36)	31.1 (73)	17.2 (5)
	Have no income	18.9 (284)	16.0 (86)	15.8 (27)	24.4 (88)	16.7 (5)	21.4 (30)	17.4 (41)	24.1 (7)
	Monthly salary	16.9 (254)	19.3 (104)	15.8 (27)	18.3 (66)	16.7 (5)	15.7 (22)	11.5 (27)	10.3 (3)
	Sale of farm produce	7.8 (117)	5.6 (30)	7.0 (12)	8.9 (32)	10.0 (3)	10.0 (14)	10.6 (25)	3.4 (1)
	Other	5.4 (81)	4.3 (23)	8.2 (14)	5.8 (21)	6.7 (2)	4.3 (6)	5.1 (12)	10.3 (3)
Mode of transport used today	Total	**1481**	**526**	**169**	**358**	**29**	**137**	**234**	**28**
	Public transport	78.3 (1159)	83.5 (439)	75.7 (128)	66.8 (239)	93.1 (27)	88.3 (121)	76.1 (178)	96.4 (27)
	Walking	19.5 (289)	14.8 (78)	21.3 (36)	30.2 (108)	6.9 (2)	10.9 (15)	21.4 (50)	0.0 (0)
	Personal car	1.9 (28)	1.3 (7)	3.0 (5)	2.2 (8)	0.0 (0)	0.7 (1)	2.6 (6)	3.6 (1)
	Other	0.3 (5)	0.4 (2)	0.0 (0)	0.8 (3)	0.0 (0)	0.0 (0)	0.0 (0)	0.0 (0)
Number that left young children (aged less than about 6 years) at home	Total	**1492**	**533**	**169**	**360**	**30**	**138**	**233**	**29**
	Yes	37.7 (563)	34.5 (184)	40.8 (69)	38.9 (140)	33.3 (10)	34.1 (47)	43.8 (102)	37.9 (11)
	No	62.3 (929)	65.5 (349)	59.2 (100)	61.1 (220)	66.7 (20)	65.9 (91)	56.2 (131)	62.1 (18)
Number who had to ask someone to accompany them	Total	**1510**	**537**	**174**	**363**	**30**	**143**	**233**	**30**
	Yes	9.9 (149)	7.8 (42)	12.6 (22)	11.8 (43)	6.7 (2)	7.7 (11)	11.6 (27)	6.7 (2)
	No	90.1 (1361)	92.2 (495)	87.4 (152)	88.2 (320)	93.3 (28)	92.3 (132)	88.4 (206)	93.3 (28)

Table 2 shows the resources used by participants. Overall, 1464 of 1620 (90.4%) participants with a single condition and 619 of 653 (94.8%) with 2 or all 3 conditions were on treatment for their health conditions. Of the diagnostic tests, the most common resources were fasting blood glucose and the plasma viral load.

**Table 2:** Resource use description showing the proportion of individuals who were on medication and the number of laboratory tests performed during the study follow-up

Resource	HIV	Diabetes	Hypertension	HIV + diabetes	HIV + hypertension	Diabetes + hypertension	HIV + diabetes + hypertension
**Number at study end**	**795**	**288**	**537**	**44**	**245**	**316**	**48**
**Medication,***n* (%)							
No medication	76 (9.6%)	31 (10.8%)	49 (9.1%)	1 (2.3%)	18 (7.3%)	14 (4.4%)	1 (2.1%)
On medication	719 (90.4%)	Overall, 257 (89.2%)	Overall, 488 (90.9%)	Both drugs, 29 (65.9%)	Both drugs, 131 (53.5%)	Both drugs, 239 (75.6%)	All drugs, 26 (54.2%)
		1 drug, 104 (36.1%)	1 drug, 115 (21.4%)	ART only, 10 (22.7%)	ART only, 87 (35.5%)	Diabetic only, 40 (12.7%)	ART only, 8 (16.7%)
		2 drugs, 144 (50.0%)	2 drugs, 282 (52.5%)	Diabetic only, 4 (9.1%)	Hypertensive only, 9 (3.7%)	Hypertensive only, 23 (7.3%)	Diabetic only, 1 (2.1%)
		3 drugs, 9 (3.1%)	3 drugs, 91 (17.0%)				Hypertensive only, 2 (4.2%)
							ART and diabetic, 6 (12.5%)
							ART and hypertensive, 3 (6.3%)
							Hypertensive and diabetic, 1 (2.1%)
**Diagnostics,***n* (%)^a^							
Viral load test	597 (75.1)	0 (0.0)	0 (0.0)	34 (77.3)	160 (65.3)	0 (0.0)	31 (64.6)
CD4 count test	87 (10.9)	0 (0.0)	0 (0.0)	2 (4.5)	23 (9.4)	0 (0.0)	3 (6.3)
HbA1c test	7 (0.9)	171 (59.4)	4 (0.7)	22 (50.0)	2 (0.8)	130 (41.1)	22 (45.8)
Fasting blood sugar test	33 (4.2)	273 (94.8)	45 (8.4)	34 (77.3)	10 (4.1)	300 (94.9)	42 (87.5)
Random blood sugar test	48 (6.0)	104 (36.1)	42 (7.8)	13 (29.5)	12 (4.9)	130 (41.1)	16 (33.3)
Haemoglobin test	41 (5.2)	7 (2.4)	5 (0.9)	2 (4.5)	12 (4.9)	2 (0.6)	3 (6.3)
Cholesterol test	6 (0.8)	34 (11.8)	28 (5.2)	2 (4.5)	6 (2.4)	26 (8.2)	1 (2.1)
Creatinine test	14 (1.8)	25 (8.7)	19 (3.5)	3 (6.8)	4 (1.6)	22 (7.0)	1 (2.1)
Urine test	7 (0.9)	11 (3.8)	15 (2.8)	2 (4.5)	5 (2.0)	17 (5.4)	2 (4.2)
Renal and liver function test	4 (0.5)	16 (5.6)	20 (3.7)	3 (6.7)	13 (6.1)	41 (13.0)	3 (6.3)
Other tests^b^	73 (9.2)	63 (21.9)	62 (11.5)	14 (31.1)	43 (20.2)	62 (19.7)	6 (12.5)

^a^Number of patients with at least a test performed during the follow-up period

^b^Other tests include tests such as pregnancy test, syphilis, malaria test, etc.

### Service use and costs of integrated care services for HIV infection, diabetes and hypertension

The mean health service cost per participant per month based on their individual resources used, assuming a full month’s patient supply of drugs and diagnostics, and assigning the overheads and personnel costs calculated for the people living with HIV infection to those living with diabetes and hypertension are shown in Table 3. In this approach, the costs of medications and of diagnostic tests per patient per month were higher for HIV infection than the additions of costs for diabetes or hypertension in individuals with single conditions (HIV infection alone, diabetes alone and hypertension alone). For participants who had multi-morbidity, their monthly drug and diagnostic costs were simply the addition of drug and diagnostic costs of individual conditions. Facility-based personnel costs were similar across all conditions.

**Table 3:** The monthly health service cost per patient of managing individuals with one, two and three conditions in an integrated one-stop clinic in Tanzania and Uganda. The mean costs and 95% confidence intervals reported are in 2020 US$

Cost component	HIV only	Diabetes	Hypertension	HIV + diabetes^1#^	HIV + hypertension^2#^	Diabetes + hypertension^3#^	HIV + diabetes + hypertension^4^
**Total monthly cost**	$34.21 (32.92, 35.59)	$18.28 (17.52, 19.04)	$18.60 (18.13, 19.08)	$39.11 (33.99, 44.33)	$32.18 (30.35, 34.07)	$22.65 (21.86, 23.43)	$36.38 (31.83, 41.07)
**Personnel**	$7.36 (7.21, 7.52)	$7.34 (7.08, 7.61)	$8.15 (7.99, 8.32)	$6.71 (6.15, 7.26)	$6.62 (6.36, 6.90)	$7.72 (7.49, 7.94)	$6.37 (5.80, 6.95)
**Medication**	$12.21 (11.60, 12.83)	$2.11 (1.82, 2.41)	$3.64 (3.43, 3.86)	$18.54 (15.04, 22.36)^a^	$14.12 (12.98, 15.30)^b^	$4.99 (4.53, 5.46)^c^	$16.98 (13.94, 20.45)^d^
**Diagnostics**	$9.24 (8.30, 10.35)	$3.64 (3.34, 3.97)	$0.45 (0.32, 0.61)	$9.65 (7.31, 11.99)	$6.81 (6.08, 7.54)	$3.87 (3.49, 4.23)	$9.07 (7.08, 11.08)
**Overheads**	$5.39 (5.16, 5.63)	$5.19 (4.80, 5.59)	$6.36 (6.04, 6.67)	$4.21 (3.61, 4.86)	$4.64 (4.33, 4.95)	$6.07 (5.72, 6.42)	$3.97 (3.39, 4.60)
Administration	$3.61 (3.39, 3.82)	$3.69 (3.36, 4.03)	$4.56 (4.27, 4.85)	$2.43 (2.03, 2.95)	$2.78 (2.52, 3.06)	$4.19 (3.89, 4.49)	$2.25 (1.87, 2.73)
Equipment and furniture	$0.38 (0.35, 0.41)	$0.34 (0.29, 0.38)	$0.40 (0.36, 0.43)	$0.44 (0.31, 0.56)	$0.39 (0.34, 0.44)	$0.51 (0.46, 0.56)	$0.38 (0.27, 0.50)
Rental space	$1.41 (1.37, 1.44)	$1.16 (1.11, 1.22)	$1.40 (1.36, 1.43)	$1.34 (1.16, 1.51)	$1.47 (1.40, 1.53)	$1.37 (1.32, 1.42)	$1.34 (1.16, 1.51)

^a^The mean cost for antiretroviral therapy was $16.92 (95% CI 13.22, 20.84) and that of diabetes drugs was $1.62 (95% CI 0.96, 2.39)

^b^The mean cost for antiretroviral therapy was $11.88 (95% CI 10.81, 12.99) and that of hypertension drugs was $2.24 (95% CI 1.91, 2.59)

^c^The mean cost of diabetes medication was $1.75 (95% CI 1.44, 2.09) and that of hypertension medication was $3.24 (95% CI 2.95, 3.54)

^d^The mean cost for antiretroviral therapy was $13.26 (95% CI 10.62, 16.15), hypertension drugs was $2.53 (95% CI 1.78, 3.35) and that of diabetes drugs was $1.19 (95% CI 0.64, 1.94)

^1^The cost of managing an individual with HIV and diabetes was − $13.39 (95% CI − 18.69, − 8.02) cheaper than managing two individuals with single conditions of HIV and diabetes, i.e., cost reductions of 25.5% (95% CI 15.4%, 35.5%)

^2^The cost of managing an individual with HIV and hypertension was − $20.63 (95% CI − 23.00, − 18.31) cheaper than managing two individuals with single conditions of HIV and hypertension, i.e., cost reductions of 39.1% (95% CI 35.1%, 42.9%)

^3^The cost of managing an individual with diabetes and hypertension was − $14.24 (95% CI − 15.42, − 13.05) cheaper than managing two individuals with single conditions of diabetes and hypertension, i.e., cost reduction of 38.6% (95% CI 36.0%, 41.1%)

^4^The cost of managing an individual with three conditions HIV, diabetes and hypertension is − $34.72 (95% CI − 39.53, − 29.75) cheaper than the cost of managing three individuals with single conditions of HIV, diabetes and hypertension, i.e., cost reductions of 48.8% (95% CI 42.1%, 55.3%). The overall cost of managing individuals with multi-morbidity (2 or 3 conditions) is − $20.74 (95% CI − 37.94, − 9.83) cheaper than the cost of two or more individuals with single conditions, i.e., cost reductions of 38.0% (95% CI 18.8%, 53.1%)

^#^The average additional cost per month of managing: (i) diabetes in an HIV-infected individual is $4.89 (95% CI − 0.36, 10.22), (ii) hypertension in HIV-infected individual is − $2.03 (95% CI − 4.33, 0.27) and (iii) hypertension in diabetic individuals is $4.37 (95% CI 3.27, 5.49)

The added monthly costs of managing either hypertension or diabetes among participants living with HIV infection were − $2.03 (95% CI − 4.33, 0.27) and +$ 4.89 (95% CI − 0.36, 10.22) per participant, respectively, compared to managing HIV only (Table 3). For people living with diabetes, the additional cost of managing hypertension was $4.37 (95% CI 3.27, 5.49) per participant per month, compared to managing diabetes only.

The mean health service costs of managing two conditions in one participant were $39.11 (95% CI 33.99, 44.33), $32.18 (95% CI 30.35, 34.07) and $22.65 (95% CI 21.86, 23.43) for HIV and diabetes, HIV and hypertension, diabetes and hypertension per month, respectively. These costs were 34.4% (95% CI 17.9%, 41.9%) lower than managing two conditions separately in two different participants. The cost of managing an individual with all three conditions was $36.38 (95% CI 31.83, 41.07) per month, which was 48.8% (95% CI 42.1%, 55.3%) lower than managing 3 conditions separately in three different participants.

### Participant-related household expenditure

The overall mean participant-related cost was $7.33 (95% CI 3.70, 15.86) per visit, and this comprised consultation costs, transport costs, medication costs, lost labour and other out-of-pocket costs (Table 4). The mean transport costs were $1.44 (95% CI 0.81, 2.18) for a return trip to health facilities per visit in the two countries, which amounts to 4.5% (95% CI 2.1, 9.6) of the participant’s total household income. Mean medication costs of $2.25 (95% CI 0.00, 7.55) per participant per month were the main drivers of patient-related costs, mainly for participants without HIV. Overall, participant-related costs per visit, constituted 11.7% (95% CI 7.3, 22.1) of the monthly total household earnings.

**Table 4:** Income and health cost for participants with single, two and three conditions attending an integrated one-stop clinic in Uganda and Tanzania. The mean costs and 95% confidence intervals reported are in 2020 US$

Cost component	HIV only	Diabetes	Hypertension	HIV + diabetes	HIV + hypertension	Diabetes + hypertension	HIV + diabetes + hypertension
Consultation costs	$0.20 (0.12, 0.30)	$0.81 (0.45, 1.27)	$0.27 (0.13, 0.44)	$0.68 (0.00, 1.58)	$0.08 (0.00, 0.19)	$0.50 (0.26, 0.77)	$0.16 (0.00, 0.46)
Treatment costs	$0.05 (0.00, 0.12)	$2.64 (0.96, 5.26)	$0.67 (0.15, 1.53)	$1.01 (0.00, 2.72)	$0.01 (0.00, 0.04)	$0.96 (0.21, 2.21)	$0.00 (0.00, 0.00)
Medication costs	$0.03 (0.00, 0.08)	$4.81 (2.49, 7.53)	$1.69 (0.81, 2.80)	$4.40 (0.00, 10.58)	$0.14 (0.00, 0.38)	$3.78 (1.30, 7.25)	$0.90 (0.00, 2.45)
Transport costs	$1.37 (1.24, 1.52)	$1.16 (0.96, 1.39)	$0.86 (0.75, 0.97)	$1.85 (1.29, 2.43)	$1.66 (1.33, 2.03)	$1.46 (1.17, 1.79)	$1.73 (1.28, 2.22)
Transport costs for other persons	$0.07 (0.04, 0.10)	$0.15 (0.07, 0.24)	$0.09 (0.06, 0.13)	$0.05 (0.00, 0.14)	$0.10 (0.03, 0.20)	$0.22 (0.07, 0.42)	$0.12 (0.00, 0.30)
Lost labour costs	$0.92 (0.73, 1.13)	$0.90 (0.66, 1.18)	$0.50 (0.38, 0.67)	$0.70 (0.33, 1.19)	$0.82 (0.59, 1.08)	$0.74 (0.50, 1.06)	$0.95 (0.39, 1.83)
Other costs (food)	$1.56 (1.39, 1.76)	$1.85 (1.53, 2.21)	$1.57 (1.32, 1.88)	$1.48 (0.82, 2.35)	$1.30 (1.03, 1.63)	$2.02 (1.53, 2.63)	$1.34 (0.95, 1.80)
**Total monthly patient-related costs**^a^	$4.20 (3.82, 4.61)	$12.32 (8.13, 17.48)	$5.66 (4.15, 7.61)	$10.17 (3.79, 18.75)	$4.11 (3.47, 4.84)	$9.68 (6.10, 14.78)	$5.20 (3.71, 7.02)
**Average monthly household income**	$75.15 (65.34, 86.95)	$125.4 (73.55, 214.9)	$89.43 (70.86, 111.2)	$81.81 (52.26, 115.2)	$79.09 (59.68, 101.3)	$95.20 (67.41, 136.0)	$76.23 (42.66, 117.0)
**Total integrated costs**^b^	$38.38 (37.03, 39.83)	$25.80 (23.42, 28.88)	$22.57 (21.65, 23.68)	$44.88 (39.17, 50.81)	$36.15 (34.20, 38.11)	$28.55 (26.93, 30.61)	$40.67 (36.04, 45.44)
**% of transport costs as a function of monthly household income**^c^	4.4% (3.7, 5.2)	3.0% (2.1, 4.0)	2.5% (2.0, 3.2)	6.9% (2.4, 13.2)	6.2% (4.3, 8.6)	4.4% (3.2, 5.8)	3.8% (2.1, 5.9)
**% of total patient-related costs as a function of monthly income**^d^	9.3% (8.3, 10.5)	12.2% (9.7, 14.8)	9.2% (7.5, 11.1)	17.8% (9.3, 27.5)	11.7% (8.9, 14.8)	12.2% (10.0, 14.6)	9.3% (5.8, 13.4)
**% of total patient-related costs as a function of total integrated cost**^e^	10.9% (10.0, 12.1)	47.5% (34.0, 61.8)	25.0% (18.9, 32.4)	22.5% (8.9, 39.9)	11.4% (9.7, 13.3)	33.7% (22.3, 48.8)	12.8% (9.2, 17.4)

^a^Average patient costs for all participants combined is $7.33 (95% CI 3.70, 15.86) with transport and medication costs of $1.44 (95% CI 0.81, 2.18) and $2.25 (95% CI 0.00, 7.55), respectively

^b^Total integrated cost is the sum of total health service cost (Table 3) and total patient-related costs minus patient-paid medication costs to avoid duplication

^c^The proportion of transport cost as a function of the monthly household income per health care visit for patients with single conditions was 3.3% (95% CI 2.1, 4.9) and 5.3% (95% CI 2.4, 10.7) for patients with multiple conditions. The overall proportion of transport costs as a function of monthly household income was 4.5% (95% CI 2.1, 9.6). Computed only for participants with a household income

^d^The proportion of total out-of-pocket expenditure as a function of the monthly household income per health care visit for patients with single conditions was 10.3% (95% CI 7.9, 14.2) and 12.7% (95% CI 6.8, 24.0) for patients with multiple conditions. The overall proportion of patient-related costs as a function of monthly household income was 11.7% (95% CI 7.3, 22.1). Computed only for participants with a household income

^e^The overall proportion of total patient-related costs as a function of total integrated cost was 23.4% (95% CI 9.9, 54.3)

Participants also reported losing earnings from visiting the facilities. This loss was nearly as much as their two-way transport costs (Supplementary Table A4 in the Additional file 1). The mean travelling time to reach the facilities was 45 min in Tanzania and 30 min in Uganda, which translates to a mean of $0.78 (95% CI 0.41, 1.31) per return trip economic cost for loss of earnings or productivity.

All in all, out-of-pocket healthcare expenditure per health care visit comprised 23.4% (95% CI 9.9, 54.3) of the total cost of integrated care (i.e., the sum of health service and patient-related costs).

### Staff- and patient-related time and time costs per visit

In our estimates, we used the observed time spent by health care staff members to provide patient care and calculated the involved costs per visit (Table 5). Clinicians spent the most time devoted to participant care, with a mean 15.3 (95% CI 11.4, 20.3) min per participant. The staff time, hence, staff cost for providing care to individuals with multiple conditions was similar or was slightly higher but not significantly different from the staff cost of caring for individuals with a single condition. Clinicians took a mean time of 15.3 (95% CI 11.9, 19.6) min per participant with a single condition and 15.4 (95% CI 11.1, 20.9) min for a participant with multiple conditions, nurses a mean time of 11.1 (95% CI 6.3, 16.2) and 13.8 (95% CI 5.4, 33.7) min and laboratory technicians 9.2 (95% CI 4.3, 17.3) and 11.5 (95% CI 7.0, 22.0) min, respectively.

**Table 5:** Health worker and patient cost by disease condition based on measured time involvement (US$ 2020). Mean costs and 95% confidence intervals are given in parentheses

Provider	HIV only	Diabetes	Hypertension	HIV + diabetes	HIV + hypertension	Diabetes + hypertension	HIV + diabetes + hypertension
Clinician	$1.70 (1.49, 2.08)	$1.96 (1.49, 2.37)	$1.59 (1.32, 2.00)	$1.92 (1.68, 2.18)	$1.82 (1.41, 2.59)	$1.45 (1.16, 1.69)	$2.25 (1.52, 2.81)
Technician	$0.43 (0.33, 0.60)	$0.79 (0.55, 1.16)	$0.41 (0.20, 0.61)	$0.60 (0.54, 0.71)	$0.76 (0.55, 0.93)	$0.59 (0.43, 0.96)	$1.05 (0.55, 1.61)
Nurse	$0.54 (0.35, 0.71)	$0.56 (0.36, 0.74)	$0.44 (0.26, 0.65)	$0.48 (0.36, 0.55)	$0.99 (0.49, 1.58)	$0.32 (0.20, 0.45)	$0.49 (0.42, 0.56)
Pharmacist	$0.34 (0.23, 0.43)	$0.58 (0.30, 0.78)	$0.50 (0.41, 0.63)	$0.37 (0.15, 0.76)	$0.49 (0.27, 0.99)	$0.34 (0.15, 0.51)	$0.16 (0.12, 0.21)
Triage	$0.93 (0.56, 1.37)	$0.96 (0.62, 1.44)	$0.71 (0.47, 0.90)	$0.41 (0.18, 0.61)	$0.92 (0.35, 2.35)	$0.50 (0.33, 0.75)	$1.72 (0.30, 2.60)
Patient	$1.42 (0.85, 2.42)	$1.26 (1.13, 1.47)	$1.86 (1.35, 2.91)	$1.44 (0.95, 2.13)	$1.05 (0.67, 1.37)	$1.57 (1.17, 2.42)	$1.59 (0.99, 2.63)

The mean total time participants took at the facility (i.e., from when they registered their arrival at the health facility, until they left the facility care) was 145.8 (95% CI 86.0, 184.7) min per patient. We observed that this time estimate was not significantly different for a visit among participants with a single condition (mean time of 150.3 (95% CI 126.3, 176.6) min) and multiple conditions (mean time of 142.4 (95% CI 74.6, 189.8) min).

This observed time translates to a mean loss of participant productivity of $1.42 (95% CI 0.85, 2.42) per participant per visit based on the estimated participant’s earnings per month (Table 4)

Overall, the lowest percentage reduction in monthly income occurred among people with HIV only and those with only hypertension, i.e., 9.3% (8.3, 10.5) and, respectively 9.2% (7.5, 11.1). This was higher among people with combinations of conditions, HIV and diabetes, HIV and hypertension, and hypertension and diabetes. This was respectively 17.8% (9.3, 27.5), 11.7% (8.9, 14.8) and 12.2% (10.0, 14.6). Hence, the latter are the groups that benefit most from one-stop clinics and become less financially challenged.

### Benchmark efficiency of service provision among integrated health facilities

To assess the efficiency changes of service delivery through the integration of services, we used the staff numbers, the number of diagnostic tests and drug availability collected at each facility (grouped as HIV clinics, NCD clinics and outpatient departments) as key quality indicators for each facility performance. We show that there was a varying level of efficiency in the service delivery among some sites (Table 6), as the mean efficiency score of a scale from 0.0 to 1.0 was 0.86 (range for all 10 facilities, 0.30–1.00). This suggests that some facilities had room to add additional participants without the need to increase the key staff numbers and without compromising their current standard or quality of care. So some HIV clinics and outpatient departments had capacity to accommodate extra participants without extra staff costs, whereas none of NCD clinics had such extra capacity (Table 6). We included the overcapacity estimates based on the DEA to adjust the prevalence-based budget projections downwards.

**Table 6:** Relative facility efficiency scores on the delivery of integrated care for people with HIV, diabetes and/or hypertension. Values are given as means and ranges in parentheses

Clinic	Inputs			Outputs			Relative Efficiency
	Staff			Drug availability^a^	Staff workload (per 1000 patient visits)^b^	Number of laboratory tests per patient^c^	
	Doctors	Nurses	Other staff				
Overall	3 (0–8)	4 (1–11)	6 (2–16)	0.82 (0.01–1.00)	0.39 (0.13–1.36)	0.61 (0.00–1.81)	0.86 (0.30–1.00)
HIV	3 (2–5)	3 (2–6)	4 (2–6)	1.00 (1.00–1.00)	0.30 (0.23–0.44)	0.91 (0.25–1.81)	0.89 (0.35–1.00)
NCD	1 (0–3)	2 (1–5)	6 (2–14)	0.68 (0.40–1.00)	0.48 (0.19–1.11)	0.30 (0.09–0.63)	1.00 (1.00–1.00)
OPD	5 (3–8)	7 (4–11)	9 (3–16)	0.60 (0.01–1.00)	0.47 (0.13–1.36)	0.33 (0.00–1.47)	0.68 (0.30–1.00)

*NCD* non-communicable disease, *OPD* outpatient department

Other staff: this includes laboratory personnel and pharmacists

^a^Drug availability — defined as the proportion of patients who get free drugs from the facilities before stock-outs occur

^b^Staff workload — the number of full-time staff divided by the number of daily patient visits

^c^Number of laboratory tests per patient — the number of laboratory tests divided by the number of patients’ visits

## Discussion

This detailed socio-economic study shows that integrated one-stop management of chronic conditions — specifically HIV infection, diabetes and hypertension — could reduce costs per participant between 34 to almost 50%, compared to managing each condition separately and hence is a highly cost-saving approach in African settings. The savings are mostly among people presenting with multiple conditions, who are financially most affected. Integration lowered the mean health service cost per visit through a one-time use of both personnel and capital resources. Participants with multiple conditions made one trip every 1 to 3 months to a health facility under the integrated care model. This is more convenient and less costly than two or three visits as is the case across sub-Saharan Africa now, if participants are treated in vertical stand-alone clinicals for each condition. This results in huge out-of-pocket savings for them and reduced duplication of clinical management activities at the health service.

At the health service, the added cost of managing multi-morbidity is essentially determined by the additional costs of drugs and diagnostics for the second or third chronic condition. Savings are realised through reductions in health worker time and in patients’ time, per participant. The time taken by health care workers, including doctors and nurses, appeared to be similar whether they were managing one condition or all three. This is not uncommon in clinical practice. It might be possible that clinical staff, working under resource constraints, allocate a fixed amount of time per participant and so did not need to give more time or attention to the multiple conditions in the absence of specific complications. Our clinical findings on a one-year follow-up [12] suggest that people with multi-morbidity had better outcomes in terms of HIV viral suppression, glycaemia and blood pressure control than people with single diseases. It is more likely, that key elements of the management of chronic conditions — clinical monitoring for disease progression and promoting drug adherence — are similar for these chronic diseases and one clinical consultation more effectively covers all of these conditions, allowing consultation time to be saved, more patients to be served, and better quality care to be delivered as indicated by the better clinical outcomes [12].

A major challenge in the management of diabetes and hypertension in Africa has been the limited and erratic supply of drugs and diagnostics [28]. Our study shows that the costs for both conditions combined are still lower than the costs of managing HIV infection. Our HIV costs were like those derived in an earlier study [29]. In the prevalence-based budget projection for the year 2025, we considered demographic changes as well disease-specific frailty in surviving all participant categories. It shows savings of 21.6% from the integrated management of the three conditions included (see annex). Given that far more people in Africa today die from the complications associated with diabetes and hypertension than with HIV infection, it is essential that that integrated care for multi-morbidities within country health systems is strengthened. Even then, transport and other related costs will still be high and may even be crippling for some households, as our study shows. New innovative ways of managing chronic conditions are needed, such as community care, inclusion of e-health and less frequent appointments at health facilities, as is the case for HIV infections.

**Fig. 2:**
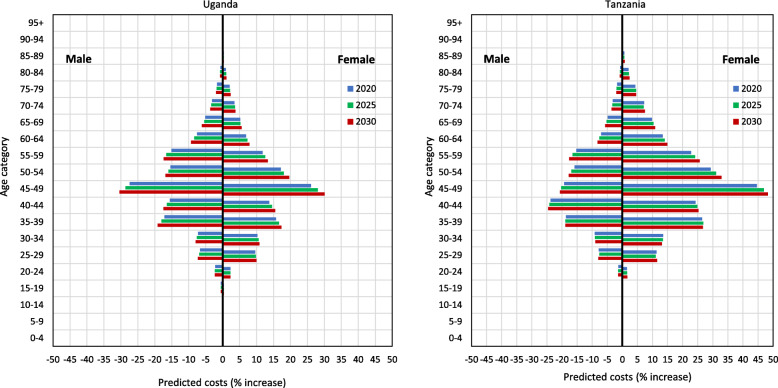
The effect of ageing and decreased survival on the total health service costs of managing people living with multiple conditions (HIV and/or diabetes and/or hypertension) in Uganda and Tanzania in years 2020, 2025 and 2030, compared to baseline

In high-income countries, about 25% of adults have multi-morbidity from chronic conditions [30, 31], while the economic burden of multi-morbidity is largely unknown. The exact burden is unknown in Africa, but non-communicable conditions have risen sharply [32, 33] alongside a continuing high burden of HIV infection and new models of chronic care management will be needed in sub-Sahara Africa. Based on existing population projections and the empirical health service costs derived in this study, we estimate the economic health service burden in people living with comorbidity (HIV and/or diabetes and/or hypertension) will go up by 21.5% (range, 19.3%, 23.4%) on average, for the next 5 years, despite the included savings of 21.6% from the integration of services. By year 2030, the economic burden may increase by 47.7% (range, 41.9%, 52.7%) compared to the current economic burden in year 2020 (Fig. 2 and Table A7 in the Additional file 1 and country statistics). Even with the ongoing changing epidemiology, most people in Africa will likely have a single chronic condition, at least for the decade to come and the costs associated with this in a vertical clinical, compared with our model, are unlikely to be appreciably different. The key question will be which models of care lead to better clinical and economic outcomes for patients with multiple conditions.

This first economic cluster-based study on real-life integrated care has some study design limitations to consider because it comprises a single-arm cohort over a 1-year follow-up duration [15, 34]. In the explorative phase of developing integrated care, we set-up this cohort without a control arm to test its acceptability to patients. Here, patients were given a choice to participate, as HIV control in a new integrated setting could be weakened due to stigma. This has guided the choice for a cohort design that has demonstrated an increase in compliance and control levels [12]. In our cohort study, a comparison group in a real-life setting (e.g., in a before-after study or a control arm) would not present the correct comparison in the absence of consistent drug supplies, as in real-life shortages in diagnostics and medicines for diabetes and hypertension control are common and patient adherence is therefore, likely, very limited. Secondly, while one may consider it unethical to withhold proven-effective treatment in control groups [35], our next follow-up study — already started — is a cluster-randomised trial based, therefore, on equipoise effects and harm of integration, pursuing a more rigorous and larger design that is needed to assess the effects of integration [15, 36].

We want to argue that real-life economic costing studies are less subject to Hawthorne effects among providers or patient participants and that the clusters are less prone to contamination: the benefits are less tangible, and hence, the estimates are less biassed. We did compare large groups of participants with a single condition to large within-cohort groups of participants with multiple conditions in both countries and consider the approach, in principle, rather robust, given the study design. Also, the follow-up period is long enough to arrive stable economic estimates of clinical resource use as patients are already diagnosed and the treatment of patients with chronic conditions tend to stabilise within six months, especially those with multiple conditions [12].

In principle, we expect some generalizable mechanisms across similar settings as the potential service effects (less programme costs per patient and less health worker cost) and effects for patients (reduction of visits) are both rather intuitive, certainly for people with multiple conditions. This will need to be proven in a larger multi-country cluster-trial, considering the existing situation and the implementation costs of the upscaled efforts. In future research, one must distinguish carefully studies addressing multi-morbidities and those addressing integration of care. The former would need individual randomisation while the latter needs cluster-randomisation at a large scale.

However, presently, one cannot say much about country differences. We need more qualitative work [14] and larger samples in the quantitative research, including step-wedge designs.

There are many factors that will influence how integrated services are implemented or the potential impact that the adoption of integration will have. As this was a feasibility study, the findings are indicative of impact, but more robust evidence is required before definitive recommendations can be made. Impact will be influenced by the prevalence of the individual conditions, organisation of health services including availability of insurance and policy directions in each country. Based on experience since the end of MOCCA, it seems that integration might be better suited to smaller health facilities with more limited resources. Larger health facilities that are delivering more specialised care or who are accepting referrals may be better suited to only implementing some aspects of integration. In Uganda, the smaller health facilities in the study have since fully integrated their services after the end of MOCCA. This has increased the provision of services, i.e., where there were two HIV clinics per week, this is now available every day. This is explored further in the MOCCA extension study. In general, in other settings, one can expect between-facility differences at baseline. Integration would probably be less impactful in small facilities where patients with different conditions are already managed through the same channels, providing one other clear mechanism towards integrated care. Ultimately, policy decisions about integrated clinics need to combine general principles with local factors.

## Conclusion

In sum, this study is a first valid exploratory approach to detail the economic costs and economies-of-scale of integrating services for chronic conditions in integrated one-stop clinics in sub-Sahara Africa. It provides much needed evidence on the probable efficiency of such models of service provision for HIV, diabetes and hypertension. Larger studies comparing directly, possibly through multiple arms directly integrated primary care with more vertical care models in a facility-randomised way will be useful to assess the overall marginal consequences, in terms of service and household impact of integrated care. The first results are certainly very promising.

In this cohort study, we have demonstrated that one-stop treatment of comorbidities is likely worthwhile and an efficient strategy enhancing financial equity in service provision for people with multiple conditions in sub-Saharan Africa settings.

## Supplementary Information

**Additional file 1: Table A1.** Unit prices (2019/2020 Ugandan and Tanzanian Shillings). **Table A2.** Resource use specifications by single, double, and triple disease. **Table A3.** Monthly programme healthcare costs (US$ 2019/20) per patient by disease and facility. This includes staff salaries including non-clinical staff such as cleaners and drivers, drugs, medical consumables and supplies, laboratory testing, medical equipment, physical infrastructure used for patient care. **Table A4.** Out-of-pocket expenses and loss of earnings (median and ranges) in 2010/2020 US$. **Table A5.** Projected costs of managing people living with HIV, diabetes, hypertension, and multi-morbidity by gender in Tanzania and Uganda. **Table A8.** Prevalence of diabetes in Tanzania and Uganda [1]. **Table A9.** Prevalence of hypertension in Tanzania and Uganda [2, 3]. **Table A10.** HIV prevalence by age and gender in Tanzania and Uganda. **Table A11.** Projected impact of aging and differences in survival on the number of people living with HIV, diabetes, hypertension, and multi-morbidity (in thousands) by gender in Tanzania and Uganda. Sample size for the time and motion observational study. **Figure A1.** The effect of aging on the increase in the number of people with combined conditions (HIV and/or diabetes and/or hypertension) compared to cases in year 2020 by gender in Uganda and Tanzania.

## Data Availability

There is an annex to the manuscript that gives details of the economic study including unit prices and the details of budget projection and data sources. Underlying data on the presented findings are available from the authors.
