# Supplementary material for: Patient and health provider costs of integrated HIV, diabetes and hypertension ambulatory health services in low-income settings — an empirical socio-economic cohort study in Tanzania and Uganda
Source: BMC Med. 2021 Sep 10;19:230. doi: 10.1186/s12916-021-02094-2 (PMC8431904; doi:10.1186/s12916-021-02094-2)
Supplement: Supplementary file 1 — Additional file 1: Table A1. Unit prices (2019/2020 Ugandan and Tanzanian Shillings). Table A2. Resource use specifications by single, double, and triple disease. Table A3. Monthly programme healthcare costs (US$ 2019/20) per patient by disease and facility. This includes staff salaries including non-clinical staff such as cleaners and drivers, drugs, medical consumables and supplies, laboratory testing, medical equipment, physical infrastructure used for patient care. Table A4. Out-of-pocket expenses and loss of earnings (median and ranges) in 2010/2020 US$. Table A5. Projected costs of managing people living with HIV, diabetes, hypertension, and multi-morbidity by gender in Tanzania and Uganda. Table A8. Prevalence of diabetes in Tanzania and Uganda [1]. Table A9. Prevalence of hypertension in Tanzania and Uganda [2, 3]. Table A10. HIV prevalence by age and gender in Tanzania and Uganda. Table A11. Projected impact of aging and differences in survival on the number of people living with HIV, diabetes, hypertension, and multi-morbidity (in thousands) by gender in Tanzania and Uganda. Sample size for the time and motion observational study. Figure A1. The effect of aging on the increase in the number of people with combined conditions (HIV and/or diabetes and/or hypertension) compared to cases in year 2020 by gender in Uganda and Tanzania. [file 12916_2021_2094_MOESM1_ESM.docx]

**Annex**

**To original article submission “Patient and provider costs of integrated HIV, diabetes, and hypertension ambulatory health services in low-income settings – an empirical socio-economic cohort study in Tanzania and Uganda”**

This annex includes referred additional supplementary tables in the text and additional results.

**Table A1.** Unit prices (2019/2020 Ugandan and Tanzanian Shillings)

| **Resource item** | **Uganda (UgX)** | **Source** | **Tanzania (TzX)** | **Source** |
| --- | --- | --- | --- | --- |
| **Laboratory, per test** |  |  |  |  |
| Urea/Creatinine | 20000 | Sinux Kiruddu | 10000 | Lancet Tanzania |
| Urinalysis | 10000 | Sinux Kiruddu | 10000 | Lancet Tanzania |
| Full blood count | 15000 | Sinux Kiruddu | 10000 | Lancet Tanzania |
| HIV rapid testing | 10000 | Sinux Kiruddu | 22900 | Lancet Tanzania |
| CD4 count | 50000 | Sinux Kiruddu | 30000 | Lancet Tanzania |
| Viral load | 250000 | Sinux Kiruddu | 120000 | Lancet Tanzania |
| Renal function test | 40000 | Sinux Kiruddu | 10000 | Lancet Tanzania |
| Liver function (ALT) test | 40000 | Sinux Kiruddu | 84900 | Lancet Tanzania |
| Haemoglobin test | 65000 | Sinux Kiruddu | 13900 | Lancet Tanzania |
| Syphilis (VDRL) test | 10000 | Sinux Kiruddu | 10000 | Lancet Tanzania |
| Malaria RDC test | 10000 | Sinux Kiruddu | 5000 | Lancet Tanzania |
| Pregnancy test | 45000 | Sinux Kiruddu | 17900 | Lancet Tanzania |
| H. Pylori test | 15000 | Sinux Kiruddu | 54900 | Lancet Tanzania |
| Hepatitis B test | 45000 | Sinux Kiruddu | 10000 | Lancet Tanzania |
| Gene Xpert | 45000 | Sinux Kiruddu | 61900 | Lancet Tanzania |
| CrAg test | 25000 | Sinux Kiruddu | 16000 | Lancet Tanzania |
| Random blood sugar test | 10000 | Sinux Kiruddu | 9900 | Lancet Tanzania |
| Fasting blood sugar test | 10000 | Sinux Kiruddu | 9900 | Lancet Tanzania |
| HBA1C test | 35000 | Sinux Kiruddu | 54900 | Lancet Tanzania |
| OGTT test | 65000 | Sinux Kiruddu | - |  |
| Lipid profile test | 40000 | Sinux Kiruddu | 54900 | Lancet Tanzania |
| **Medication** |  |  |  |  |
| ***Antiretroviral therapy per month*** |  |  |  |  |
| TDF/3TC/DTG 300mg/300mg/50mg, per 30 tablets | 32567.56 | NMS | 19866.21 | GPRM |
| AZT/3TC/NPV 300mg/150mg/200mg, per 60 tablets | 38004.63 | NMS | 23182.82 | GPRM |
| DTG 50mg, per 30 tablets | 10291.65 | NMS | 6277.91 | GPRM |
| EFV 200mg, per 30 tablets | 16430.37 | GPRM | 10022.53 | GPRM |
| RTV 100mg, per 30 tablets | 18307.84 | NMS | 11167.78 | GPRM |
| ATV/r 300mg/100mg, per 30 tablets | 60616.83 | GPRM | 36976.27 | GPRM |
| TDF/3TC/EFV 300mg/300mg/600mg, per 30 tablets | 38706.28 | NMS | 23610.83 | GPRM |
| TDF/3TC 300mg/300mg, per 30 tablets | 22275.91 | NMS | 13588.31 | GPRM |
| AZT/3TC 300mg/150mg, per 60 tablets | 39638.64 | NMS | 24179.57 | GPRM |
| LPV/r 200mg/50mg, per 120 tablets | 130908.90 | NMS | 79854.43 | GPRM |
| NVP 200mg, per 30 tablets | 23088.64 | GPRM | 14084.07 | GPRM |
| ***Diabetes treatment per month*** |  |  |  |  |
| Insulin mixtard human 100IU/ML, per 100 vials | 17861.20 | NMS | - |  |
| Glibenclamide 5mg, per 100 tablets | 3143 | NMS | - |  |
| Metformin HCL 500mg, per 100 tablets | 3576 | NMS | - |  |
| Glimepiride 2mg + Metformin 500mg | - |  | 8800 | MSD |
| Glimepiride 1mg + Metformin 500mg | - |  | 6000 | MSD |
| Losartan 50mg + Hydrochlothiazide 12.5mg | - |  | 15800 | MSD |
| Losartan 50mg + Hydrochlothiazide 5mg | - |  | 12600 | MSD |
| ***Hypertension treatment per month*** |  |  |  |  |
| Bendroflumethiazide 5mg, per 100 tablets | 25128 | NMS | 750 | MSD |
| Amlodipine 10mg, per 100 tablets | 6912 | NMS | 2872 | MSD |
| Nifedipine 5mg, per 100 tablets | 7000 | NMS | 6500 | MSD |
| Furosemide 40mg, per 1000 tablets | 11700 | NMS | 573 | MSD |
| Enalapril 10mg, per 28 tablets | 9072 | NMS | 8200 | MSD |
| Atenolol 100mg, per 1000 tablets | 26100 | NMS | 600 | MSD |
| **Antibiotics** |  |  |  |  |
| Cotrimoxazole 120mg, per 100 tablets | 7600 | NMS | - |  |
| Amoxicillin/Ampicillin 250mg capsule, per 20 capsules | 531000 | NMS | - |  |
| **Other drugs** |  |  |  |  |
| Fluconazole 200mg, per 100 tablets | 202000 | NMS | - |  |

NMS: national medical stores; GPRM: Global price reporting mechanism (WHO); CrAg: Cryptococcal antigen; OGTT: oral glucose tolerance test, HBA1C: Glycated haemoglobin A1c;

Sinux Kiruddu laboratory price list 2019

**Table A2.** Resource use specifications by single, double, and triple disease

|  | **Single disease conditions** | | | **Double disease conditions** | | | **Triple disease condition** |
| --- | --- | --- | --- | --- | --- | --- | --- |
| **Cost component** | **Diabetes** | **Hypertension** | **HIV** | **Diabetes + Hypertension** | **Diabetes + HIV** | **Hypertension + HIV** | **Diabetes + Hypertension + HIV** |
| **Staff** |  |  |  |  |  |  |  |
|  |  |  |  |  |  |  |  |
| **Medications** | Diabetes drugs | BP drugs | Antiretroviral drugs | Diabetes drugs | Diabetes drugs | BP drugs | Diabetes drugs |
|  |  |  |  | BP drugs | ART + HIV-related complications | ART + HIV-related complications | BP drugs |
|  |  |  |  |  |  |  | ART + HIV-related complications |
|  |  |  |  |  |  |  |  |
| **Laboratory tests** | RBS, FBS, LFT, RFT | LFT, RFT, Lipids, RBS, | CD4 count, VL, FBC | RBS, FBS, LFT, RFT, | RBS, FBS, LFT, RFT, | LFT, RFT, CD4 count | RBS, FBS, LFT, RFT, Lipids, |
|  | HBA1C, Lipids, FBP | Creatinine, FBS, | Haemoglobin, RBS, | HBA1C, Lipids, BUN, | HBA1C, CD4 count, VL, | VL, Lipids | HBA1C, CD4 count, VL |
|  | Creatinine, BUN, | MRDT, Urinalysis | BUN, creatinine, | Urinalysis, creatinine | Lipids, |  |  |
|  | Urinalysis |  | HBA1C, haemoglobin |  |  |  |  |
| **Overhead costs** |  |  |  |  |  |  |  |
| Administration | NCD administration | NCD administration | CTC administration | NCD administration | NCD or CTC admin | NCD or CTC admin | NCD or CTC administration |
|  |  |  |  |  |  |  |  |
| Rental space | NCD space | NCD space | CTC space | NCD space | NCD or CTC space | NCD or CTC space | NCD or CTC space |
|  |  |  |  |  |  |  |  |
| Furniture | NCD furniture | NCD furniture | CTC furniture | NCD furniture | NCD or CTC furniture | NCD or CTC furniture | NCD or CTC furniture |
|  |  |  |  |  |  |  |  |
| Equipment | Glucometers, | BP machines | Weighing scales | Glucometers, | Glucometers, | BP machines | Glucometers, |
|  | Weighing scales | Weighing scales | Stethoscopes | Weighing scales | Weighing scales | Weighing scales | Weighing scales |
|  | Stethoscopes | Stethoscopes | BP machines | Stethoscopes | Stethoscopes | Stethoscopes | Stethoscopes |
|  | BP machines |  |  | BP machines | BP machines |  | BP machines |

Administration includes utilities, transport, and clinical consumables

LFT: Liver function test; RFT: Renal function test; BP: blood pressure; ART: antiretroviral therapy; FBS: Fasting blood sugar; RBS: random blood sugar; VL: Viral load; NCD: Non-communicable diseases; CTC: Chronic treatment centre; FBC: Full blood count;

Staff time, administration costs, facility rental costs, furniture costs, equipment costs can provide economies of scale during integration for individuals with two or more conditions.

**Table A3.** Monthly programme healthcare costs (US$ 2019/20) per patient by disease and facility. This includes staff salaries including non-clinical staff such as cleaners and drivers, drugs, medical consumables and supplies, laboratory testing, medical equipment, physical infrastructure used for patient care.

| **Type of cost** | **Type of care** | **Tanzania**  Mean (range) | **Uganda**  Mean (range) |
| --- | --- | --- | --- |
| **Personnel** | HIV patients | $5.34 (3.44 - 9.50) | $8.65 (7.02 - 10.26) |
|  | Diabetes patients | $4.19 (2.99 - 6.06) | $6.91 (1.32 - 15.86) |
|  | Hypertension | $4.19 (2.99 - 6.06) | $6.91 (1.32 - 15.86) |
| **Medication** | HIV patients | $9.86 (9.05 - 10.73) | $9.95 (8.59 - 12.63) |
|  | Diabetes patients | $4.51 (1.05 - 5.84) | $0.72 (0.02 - 1.42) |
|  | Hypertension | $1.98 (1.34 - 2.75) | $2.80 (0.19 - 9.37) |
| **Laboratory** | HIV patients | $6.72 (3.49 - 10.46) | $16.26 (9.79 - 29.22) |
|  | Diabetes patients | $5.28 (0.23 - 13.93) | $11.07 (2.31 - 24.13) |
|  | Hypertension | $2.95 (0.00 - 6.45) | $1.61 (0.00 - 4.42) |
| **Overheads** |  |  |  |
| **Administration** | HIV patients | $1.54 (0.29 - 2.27) | $5.16 (0.80 - 10.08) |
|  | Diabetes patients | $1.11 (0.46 - 2.27) | $4.46 (0.11 - 10.61) |
|  | Hypertension | $1.11 (0.46 - 2.27) | $4.46 (0.11 - 10.61) |
| **Rental space** | HIV patients | $1.01 (0.49 - 1.98) | $1.67 (1.53 - 1.93) |
|  | Diabetes patients | $1.37 (0.53 - 2.27) | $3.56 (0.63 - 10.80) |
|  | Hypertension | $1.37 (0.53 - 2.27) | $3.56 (0.63 - 10.80) |
| **Equipment and furniture** | HIV patients | $0.13 (0.00 - 0.26) | $0.60 (0.02 - 1.18) |
|  | Diabetes patients | $0.07 (0.00 - 0.29) | $1.62 (0.93 - 3.35) |
|  | Hypertension | $0.06 (0.00 - 0.29) | $1.60 (0.93 - 3.33) |
|  |  |  |  |
| **Total** | HIV patients | $24.61 (21.56 - 33.18) | $42.30 (36.00 - 55.03) |
|  | Diabetes patients | $16.53 (6.21 - 24.81) | $28.34 (20.26 - 38.04) |
|  | Hypertension | $11.67 (6.13 - 14.51) | $20.94 (17.34 - 27.85) |

**Table A4**. Out-of-pocket expenses and loss of earnings (median and ranges) in 2010/2020 US$.

| **Component** | **Tanzania**  **Median (range)** | **Uganda**  **Median (range)** |
| --- | --- | --- |
| **Total earnings last month** | $38.70 (0.00-1290.00) | $40.50 (0.00-1080.00) |
| **Previous visits health care expenses** |  |  |
| Number of health facility visits in the last 3 months | 2.00 (0.00-14.00) | 2.00 (0.00-90.00) |
|  |  |  |
| Total medical costs last month | $10.75 (0.34-1032.00) | $8.10 (0.14-661.50) |
| Hypertensive drugs | $10.75 (0.65-64.50) | $7.56 (0.54-270.00) |
| Diabetes drugs | $15.70 (1.72-107.50) | $8.10 (0.81-108.00) |
| Antiretroviral drugs | $4.30 (4.30-5.16) | $1.49 (0.41-8.10) |
| Additional exacerbations | $4.30 (0.34-86.00) | $5.40 (0.14-661.50) |
| Hypertensive and diabetes drugs | $38.70 (17.20-1032.00) | $18.36 (2.70-135.00) |
|  |  |  |
| Total nonmedical costs last month | $1.72 (0.13-129.00) | $10.80 (0.14-540.00) |
| Money spent on transport last month | $1.20 (0.17-86.00) | $1.35 (0.00-135.00) |
| Money spent by person accompanying you last month | $1.29 (0.26-17.20) | $2.70 (0.27-67.50) |
| **Current visit health care expenses** |  |  |
| Total consultation fees paid today | $4.30 (0.43-30.10) | $0.00 |
| Treatment costs paid today | $10.32 (0.43-193.50) | $2.03 (1.35-16.20) |
| Medication costs paid today | $18.06 (0.86-181.89) | $4.05 (1.35-43.20) |
|  |  |  |
| Money spent on transport today | $0.86 (0.17-21.50) | $1.08 (0.14-18.90) |
| Money spent on transport by person accompanying you today | $0.86 (0.09-6.45) | $1.35 (0.14-15.12) |
|  |  |  |
| Money spent on other costs today | $0.90 (0.13-17.20) | $1.35 (0.14-40.50) |
| **Loss of earnings and/or productivity** |  |  |
| Time it took you to get to the facility today (minutes) | 45.00 (0.00-2000.00) | 30.00 (0.60-600.00) |
| Number of days of being ill | 1.00 (0.00-30.00) | 0.00 (0.00-90.00) |
| **Health care financing** |  |  |
| Monthly insurance premium | $15.05 (1.51-58.05) | - |

**Exchange rate used (April 2020),** 1US$ = 3 704 Ugandan shillings and 1US$ = 2 311 Tanzanian shillings

**Table A5.** Projected costs of managing people living with HIV, diabetes, hypertension, and multi-morbidity by gender in Tanzania and Uganda.

| **Country, Year** | **Gender** | **HIV** | **Diabetes** | **Hypertension** | **HIV + Diabetes** | **HIV + Hypertension** | **Diabetes + Hypertension** | **HIV + Diabetes + Hypertension** |
| --- | --- | --- | --- | --- | --- | --- | --- | --- |
| **Tanzania** |  |  |  |  |  |  |  |  |
| Year 2020 | All | USD 744,655,365 | USD 310,110,097 | USD 1,236,843,970 | USD 49,070,727 | USD 147,806,555 | USD 93,870,210 | USD 11,774,750 |
|  | Female | USD 484,351,910 | USD 176,705,138 | USD 596,825,979 | USD 33,870,427 | USD 92,679,982 | USD 53,468,756 | USD 8,015,063 |
|  | Male | USD 260,303,456 | USD 133,404,960 | USD 640,017,991 | USD 15,200,300 | USD 55,126,573 | USD 40,401,454 | USD 3,759,687 |
| Year 2025 | All | USD 881,663,683 | USD 368,564,367 | USD 1,484,283,062 | USD 58,519,780 | USD 177,848,379 | USD 113,188,344 | USD 14,219,199 |
|  | Female | USD 572,299,801 | USD 209,151,985 | USD 716,840,209 | USD 40,237,162 | USD 111,505,167 | USD 64,371,666 | USD 9,663,985 |
|  | Male | USD 309,363,881 | USD 159,412,382 | USD 767,442,853 | USD 18,282,619 | USD 66,343,212 | USD 48,816,678 | USD 4,555,215 |
| % change (2020 vs 2025) |  | 18.40 | 18.85 | 20.01 | 19.26 | 20.33 | 20.58 | 20.76 |
|  |  |  |  |  |  |  |  |  |
| Year 2030 | All | USD 1,039,916,383 | USD 439,912,708 | USD 1,774,712,644 | USD 69,628,332 | USD 212,755,626 | USD 136,323,168 | USD 17,075,944 |
|  | Female | USD 673,993,855 | USD 248,706,584 | USD 859,441,797 | USD 47,730,704 | USD 133,499,069 | USD 77,420,419 | USD 11,593,982 |
|  | Male | USD 365,922,528 | USD 191,206,124 | USD 915,270,847 | USD 21,897,628 | USD 79,256,557 | USD 58,902,748 | USD 5,481,962 |
| % change (2020 vs 2030) |  | 39.65 | 41.86 | 43.49 | 41.89 | 43.94 | 45.23 | 45.02 |
| **Uganda** |  |  |  |  |  |  |  |  |
| Year 2020 | All | USD 691,356,218 | USD 103,335,098 | USD 1,019,641,665 | USD 18,295,828 | USD 151,875,657 | USD 40,314,246 | USD 5,642,354 |
|  | Female | USD 439,167,315 | USD 45,592,626 | USD 460,916,069 | USD 9,895,091 | USD 79,145,746 | USD 16,239,668 | USD 2,646,844 |
|  | Male | USD 252,188,903 | USD 57,742,472 | USD 558,725,596 | USD 8,400,736 | USD 72,729,912 | USD 24,074,578 | USD 2,995,510 |
| Year 2025 | All | USD 831,171,568 | USD 126,418,074 | USD 1,244,528,468 | USD 22,410,677 | USD 185,727,094 | USD 49,728,255 | USD 6,954,165 |
|  | Female | USD 525,891,497 | USD 55,440,202 | USD 561,582,584 | USD 12,069,261 | USD 96,761,510 | USD 19,897,546 | USD 3,249,588 |
|  | Male | USD 305,280,071 | USD 70,977,872 | USD 682,945,884 | USD 10,341,416 | USD 88,965,584 | USD 29,830,709 | USD 3,704,577 |
| % change (2020 vs 2025) |  | 20.22 | 22.34 | 22.06 | 22.49 | 22.29 | 23.35 | 23.25 |
|  |  |  |  |  |  |  |  |  |
| Year 2030 | All | USD 999,718,746 | USD 154,964,773 | USD 1,523,216,970 | USD 27,503,088 | USD 228,212,828 | USD 61,571,849 | USD 8,595,330 |
|  | Female | USD 628,397,661 | USD 67,619,800 | USD 687,400,717 | USD 14,751,116 | USD 118,704,046 | USD 24,561,254 | USD 4,013,021 |
|  | Male | USD 371,321,086 | USD 87,344,973 | USD 835,816,253 | USD 12,751,972 | USD 109,508,782 | USD 37,010,595 | USD 4,582,309 |
| % change (2020 vs 2030) |  | 44.60 | 49.96 | 49.39 | 50.32 | 50.26 | 52.73 | 52.34 |

**Estimated future budget increases**

We also predicted the of increase of multi-conditions in the next 5 and 10 years in Uganda and Tanzania using the current prevalence of single conditions and population projections from the United Nations Population Council for the two countries <https://population.un.org/wpp/Download/Standard/Interpolated/>. The prevalence of diabetes (Table A8), hypertension (Table A9) and HIV (Table A10) were derived from literature**.** We performed targeted literature search for the prevalence of diabetes, hypertension, and HIV in the two countries and only took studies that presented results by age category and gender. Table A11 and Figure A show the predicted burden of single and multiple conditions in the two countries.

**Table A8**. Prevalence of diabetes in Tanzania and Uganda [1]

|  | Tanzania | | Uganda | |
| --- | --- | --- | --- | --- |
| Age category (years) | Male (%) | Female (%) | Male (%) | Female (%) |
| 18-24 | 0.1 (0.0 – 0.3) | 0.2 (0 – 0.4) | 0.7 (0.0 – 0.6) | 0.3 (0.0 – 0.7) |
| 25-39 | 4.3 (2.7 – 6.2) | 6.1 (2.7 – 13.3) | 2.5 (1.0 – 4.3) | 1.6 (0.6 – 2.8) |
| 40-54 | 6.0 (3.9 – 9.2) | 8.4 (6.3 – 11.0) | 2.6 (1.3 – 5.3) | 3.7 (2.2 – 6.7) |
| 55-64 | 9.2 (5.0 – 16.0) | 8.0 (5.7 – 11.9) | 8.6 (3.4 – 18.7) | 3.6 (1.4 – 9.2) |

**Table A9**. Prevalence of hypertension in Tanzania and Uganda [2, 3]

|  | Tanzania | | Uganda | |
| --- | --- | --- | --- | --- |
| Age category (years) | Male (%) | Female (%) | Male (%) | Female (%) |
| 18-24 | 14/129 (10.9) | 6/179 (3.4) | 13/107 (12.1) | 6/154 (3.9) |
| 25-34 | 19/153 (12.4) | 14/190 (7.4) | 15/109 (13.8) | 15/146 (10.3) |
| 35-44 | 24/92 (26.1) | 14/82 (17.1) | 17/59 (28.8) | 18/96 (18.8) |
| 45+ | 41/129 (31.8) | 60/139 (43.2) | 53/100 (53.0) | 69/145 (47.6) |

**Table A10.** HIV prevalence by age and gender in Tanzania and Uganda

|  | Tanzania (2016-2017) ^1^ | | Uganda (2016-17) ^2^ | |
| --- | --- | --- | --- | --- |
| Age category (years) | Female (%) | Male (%) | Male (%) | Female (%) |
| 0-4 | 0.6 | 0.2 | 0.5 | 0.6 |
| 5-9 | 0.5 | 0.5 | 0.2 | 0.6 |
| 10-14 | 0.3 | 0.3 | 0.3 | 1.1 |
| 15-19 | 1.0 | 0.4 | 0.5 | 1.8 |
| 20-24 | 3.4 | 0.9 | 1.3 | 5.1 |
| 25-29 | 5.6 | 2.3 | 3.7 | 8.5 |
| 30-34 | 8.6 | 3.9 | 5.5 | 11.4 |
| 35-39 | 11.6 | 5.6 | 9.2 | 12.9 |
| 40-44 | 11.0 | 8.4 | 10.8 | 11.9 |
| 45-49 | 12.0 | 6.8 | 14.0 | 12.8 |
| 50-54 | 9.4 | 7.4 | 9.7 | 10.5 |
| 55-59 | 9.7 | 8.0 | 8.9 | 9.4 |
| 60-64 | 6.5 | 3.6 | 5.3 | 6.9 |

^1^Tanzania HIV Impact Survey (THIS) – A population-based HIV Impact Assessment 2016-2017

^2^Uganda Population-based HIV Impact Assessment- UPHIA 2016-2017

**Table A11.** Projected impact of aging and differences in survival on the number of people living with HIV, diabetes, hypertension, and multi-morbidity (in thousands) by gender in Tanzania and Uganda.

| **Country, year** | **Gender** | **HIV** | **Diabetes** | **Hypertension** | **HIV + Diabetes** | **HIV + Hypertension** | **Diabetes + Hypertension** | **HIV + Diabetes + Hypertension** |
| --- | --- | --- | --- | --- | --- | --- | --- | --- |
| **Tanzania** |  |  |  |  |  |  |  |  |
| Year 2020 |  |  |  |  |  |  |  |  |
|  | All | 1813.9 | 1413.7 | 5541.4 | 104.6 | 382. 8 | 345.4 | 27.0 |
|  | Female | 1179.8 | 805.5 | 2674.0 | 72.2 | 240.0 | 196.7 | 18.4 |
|  | Male | 634.1 | 608.2 | 2867.4 | 32.4 | 142. 8 | 148.7 | 8.6 |
| Year 2025 | All | 2147.7 | 1680.2 | 6650.0 | 124.7 | 460.6 | 416.4 | 32.6 |
|  | Female | 1394.1 | 953.5 | 3211.6 | 85.7 | 288. 8 | 236.8 | 22.2 |
|  | Male | 753.6 | 726.7 | 3438. 4 | 39.0 | 171.8 | 179.6 | 10.4 |
| % change |  | 18.4% | 18.9% | 20.0% | 19.2% | 20.3% | 20.6% | 20.7% |
| Year 2030 | All | 2533.2 | 2005.4 | 7951.2 | 148. 4 | 551.0 | 501.6 | 39.1 |
|  | Female | 1641.8 | 1133. 8 | 3850.5 | 101.7 | 345.7 | 284.8 | 26.6 |
|  | Male | 891.4 | 871.7 | 4100.7 | 46.7 | 205.3 | 216.8 | 12.5 |
| % change |  | 39.7% | 41.9% | 43.5% | 41.9% | 43.9% | 45.2% | 44.8% |
| **Uganda** |  |  |  |  |  |  |  |  |
| Year 2020 | All | 1684.1 | 471.1 | 4568.3 | 39.0 | 393. 3 | 148.3 | 12.9 |
|  | Female | 1069.8 | 207.8 | 2065.0 | 21.0 | 205.0 | 59.7 | 6.1 |
|  | Male | 614.3 | 263.3 | 2503.3 | 18.0 | 188.3 | 88.6 | 6.8 |
| Year 2025 | All | 2024.7 | 576.3 | 5575.9 | 47.8 | 481.0 | 183.0 | 15.9 |
|  | Female | 1281.1 | 252.7 | 2516.1 | 25.8 | 250.6 | 73.2 | 7.4 |
|  | Male | 743.6 | 323.6 | 3059.8 | 22.0 | 230.4 | 109.8 | 8.5 |
| % change |  | 20.2% | 22.3% | 22.1% | 22.6% | 22.3% | 23.4% | 23.3% |
| Year 2030 | All | 2435.2 | 706.4 | 6824.5 | 58.6 | 591.0 | 226.5 | 19.7 |
|  | Female | 1530.7 | 308.3 | 3079.8 | 31.4 | 307.4 | 90.4 | 9.2 |
|  | Male | 904.5 | 398.1 | 3744.7 | 27.2 | 283. 6 | 136.1 | 10.5 |
| % change |  | 44.6% | 49.9% | 49.4% | 50.3% | 50.3% | 52.7% | 52.7% |

**Time and motion study**

An observation study that aims to quantify patient and/or caregiver time. Main outcomes are staff time and patient waiting time. We selected 30 patients per disease condition in each country. Out of these, 15 patients (say 8 females and 7 males) are “recent patients” and 15 patients should be “existing patients” (and again say 8 females and 7 males). Since we decided not to stratify by “type of health care centre”, these are sampled from a single centre. A total of 120 per country (with say 64 females and 56 males) should be sampled for the time and motion study. In each country, we have enrolled 120 patients, with 60 recently-diagnosed patients and another 60 existing patients. Numbers are balanced per disease status of patients.

Sample size for the time and motion observational study.

| **Category** | HIV | Diabetes | Hypertension | Combination | Total |
| --- | --- | --- | --- | --- | --- |
| **New patients** | 15 | 15 | 15 | 15 | 60 |
| **Existing patients** | 15 | 15 | 15 | 15 | 60 |
| **Total** | 30 | 30 | 30 | 30 | 120 |

**Figure A1**. The effect of aging on the increase in the number of people with combined conditions (HIV and/or diabetes and/or hypertension) compared to cases in year 2020 by gender in Uganda and Tanzania.

# References

1. Manne-Goehler, J., et al., *Diabetes diagnosis and care in sub-Saharan Africa: pooled analysis of individual data from 12 countries.* Lancet Diabetes Endocrinol, 2016. **4**(11): p. 903-912.

2. Kavishe, B., et al., *High prevalence of hypertension and of risk factors for non-communicable diseases (NCDs): a population based cross-sectional survey of NCDS and HIV infection in Northwestern Tanzania and Southern Uganda.* BMC Med, 2015. **13**: p. 126.

3. Mosha, N.R., et al., *Prevalence,awareness and factors associated with hypertension in North West Tanzania.* Glob Health Action, 2017. **10**(1): p. 1321279.
